# Eye Movements During Visual Speech Perception in Deaf and Hearing Children

**DOI:** 10.1111/lang.12264

**Published:** 2017-09-26

**Authors:** Elizabeth Worster, Hannah Pimperton, Amelia Ralph‐Lewis, Laura Monroy, Charles Hulme, Mairéad MacSweeney

**Affiliations:** ^1^ Institute of Cognitive Neuroscience University College London; ^2^ Deafness, Cognition, and Language Research Centre University College London; ^3^ University of Oxford

**Keywords:** deaf, hearing, lipreading, speechreading, eye gaze, eye tracking

## Abstract

For children who are born deaf, lipreading (speechreading) is an important source of access to spoken language. We used eye tracking to investigate the strategies used by deaf (*n* = 33) and hearing 5–8‐year‐olds (*n* = 59) during a sentence speechreading task. The proportion of time spent looking at the mouth during speech correlated positively with speechreading accuracy. In addition, all children showed a tendency to watch the mouth during speech and watch the eyes when the model was not speaking. The extent to which the children used this communicative pattern, which we refer to as social‐tuning, positively predicted their speechreading performance, with the deaf children showing a stronger relationship than the hearing children. These data suggest that better speechreading skills are seen in those children, both deaf and hearing, who are able to guide their visual attention to the appropriate part of the image and in those who have a good understanding of conversational turn‐taking.

## Introduction

Although speech is generally considered within the auditory domain only, visual information is very important for guiding speech perception. It is well established that congruent visual speech can enhance speech perception (Lusk & Mitchel, 2016; Mitchel & Weiss, 2014; Sumby & Pollack, 1954), while incongruent visual information can disrupt it (McGurk & MacDonald, 1976). Several studies have investigated the looking patterns people employ when watching a speaking face. In this study we examined and contrasted these patterns in young deaf and hearing children to gain insights into the processes underlying visual speech perception in these groups.

Some studies have shown that adults from Western cultures tend to look at the eyes more than the mouth during audiovisual speech perception in no noise (Smith, Giblisco, Meisinger, & Hankey, 2013; Vatikiotis‐Bateson, Eigsti, Yano, & Munhall, 1998), while others have observed a preference for the mouth over the eyes (Barenholtz, Mavica, & Lewkowicz, 2016). What appears to differentiate these two different findings is that the allocation of visual attention to the face during speech perception is highly dependent on task demands. Lansing and McConkie (1999) showed that participants allocate their attention toward the eyes when identifying emotional or prosodic information from audiovisual speech and toward the mouth when the task emphasizes segmental information. This suggests that viewers can allocate their attention to the most useful source of information. As well as being task dependent, gaze shifts as noise in the auditory stream increases, focusing further down the face to the nose and mouth (Vatikiotis‐Bateson et al., 1998). This suggests that, as speech perception becomes more difficult, perceivers are able to shift their attention to the mouth to make use of visual cues. Barenholtz et al. (2016) found that looking time to the mouth is modulated by language familiarity, with adults looking longer at the mouth when watching an unfamiliar language than a familiar one. The mouth is clearly an important source of information in audiovisual speech perception, especially when segmentation of speech is a priority or when there is uncertainty about the speech signal.

To date, however, there has been relatively little research investigating whether there is a relationship between gaze patterns and performance in audiovisual speech perception, that is, whether looking to the mouth (mouth focus) provides an advantage in audiovisual speech perception. In conditions of no noise with a single speaker, fixating away from the mouth, up to 15 degrees of eccentricity, does not affect audiovisual speech intelligibility (Yi, Wong & Eizenman, 2013). Similarly, the McGurk effect is still observed when participants fixate 10 degrees from the centre of the mouth (Paré, Richler, ten Hove, & Munhall, 2003). These results suggest that visual speech information can be accessed via peripheral vision, as suggested by Massaro (1998), calling into question why individuals fixate on the mouth in difficult perceptual situations. However, when noise is introduced by having two speakers presented side by side, speech intelligibility scores are reduced when fixating more than 2.5 degrees of eccentricity from the centre of the mouth (Yi, Wong & Eizenman, 2013). This suggests that as auditory speech perception becomes more difficult, peripheral vision is not sufficient to access supporting visual speech information from the mouth. It seems from these results that mouth focus does aid audiovisual speech perception. However, studies with hearing adults have found that mouth focus does not correlate with individuals’ ability to speechread (lipread) silent spoken sentences (Lansing & McConkie, 2003), speechread consonant‐vowel‐consonant clusters (Wilson, Alsius, Paré, & Munhall, 2016), or relate to susceptibility to the McGurk effect (Paré et al., 2003). These results are surprising given that the gaze shifts to the mouth with increasing perceptual difficulty during audiovisual speech perception. However, each of these studies only had 20 or fewer participants. Thus the lack of correlation may be due to a lack of power.

Evidence from adults suggests that visual information aids audiovisual speech perception. However, it is important to understand whether the same is true in children, whether this changes with development, and whether it affects language development. Children from 3 to 8 years old perceive the McGurk effect, although less reliably than adults (McGurk & MacDonald, 1976), as do infants as young as 4½ months old (Burnham & Dodd, 2004). Importantly, visual speech influences word‐form recognition in infants, suggesting that it plays a role in language acquisition (Weatherhead & White, 2017). Although visual information is clearly used by young infants, others have suggested that visual influence on speech perception changes throughout development. Jerger, Damian, Spence, Tye‐Murray, and Abdi (2009) showed that 5–9‐year‐old children were less distracted by visual information during a cross‐modal picture‐word matching task than both 4‐year‐olds and 10–14‐year‐olds, suggesting a U‐shaped function in visual influence.

Along with changes in visual influence on speech perception, there are also developmental changes in gaze patterns to the face. Infants younger than 8 months old show a preference for watching the eyes both during infant‐directed and adult speech (Lewkowicz & Hansen‐Tift, 2012; Smith et al., 2013). At around 8 months old, infants shift their attention to the mouth of a speaking face but return to focus on the eyes again by 12 months old (Lewkowicz & Hansen‐Tift, 2012; Pons, Bosch, & Lewkowicz, 2015). In addition, bilingual infants shift to watch the mouth earlier than monolingual infants, showing equal looking to the eyes and mouth at 4 months old, and maintain their preference for the mouth at 12 months old (Pons et al., 2015). For bilingual infants it is likely that visual information from the mouth aids differentiation between the two languages they are acquiring and thus supports language acquisition.

Although no developmental research has directly related gaze to the mouth to performance on speech perception tasks, Young, Merin, Rogers, and Ozonoff (2009) found that increased gaze to the mouth at 6 months of age predicted higher expressive language outcomes 18 months later. Overall, the developmental evidence is consistent with findings from adults that as speech perception becomes harder or more important, gaze to the mouth is prioritized. This suggests that the mouth is an important source of speech information and that children can selectively allocate their attention to make use of this information.

It is clear that visual information plays an important role in audiovisual speech perception for hearing adults and children, but it is of particular importance for individuals who are deaf. Even with a cochlear implant (CI), deaf children have reduced access to the auditory speech signal compared to hearing children. Thus they are more dependent than hearing children on speechreading to aid spoken language perception. However, some speech sounds are visually indistinguishable, such as /m/, /p/ and /b/, making it difficult to fully access the speech signal through vision alone. Despite this, speechreading ability does vary considerably between individuals in both deaf and hearing populations (Mohammed, Campbell, MacSweeney, Barry, & Coleman, 2006). Whether an individual's speechreading ability is fixed or can be improved is controversial. Deaf adults who have been deaf from an early age have equivalent or superior speechreading skills compared to hearing adults (Bernstein, Demorest, & Tucker, 2000; Mohammed et al., 2006; Pimperton, Ralph‐Lewis, & MacSweeney, 2017). However, it has been shown that deaf children do not have superior speechreading skills compared to their hearing peers (Kyle, Campbell, Mohammed, & Coleman, 2013; Kyle & Harris, 2006). In addition, a study of deaf adults with CIs indicated that later implantation may relate to higher speechreading scores (Pimperton et al., 2017). These results suggest that greater experience with and attention to speechreading can enhance speechreading proficiency.

There is huge variability in the reading ability of deaf children and adults, with many excelling. However, on average deaf children have been shown to have poorer reading skills than their hearing peers (Conrad, 1979; DiFrancesca, 1972; Qi & Mitchell, 2011; Wauters, van Bon & Tellings, 2006). Importantly, speechreading correlates with reading in deaf children (Arnold & Kopsel, 1996; Kyle, Campbell, & MacSweeney, 2016; Kyle & Harris, 2006), regardless of the child's preferred language (Kyle & Harris, 2010, 2011); across linguistic levels (single word and sentences; Kyle et al., 2016); and is a longitudinal predictor of reading development (Kyle & Harris, 2010). In addition, speechreading relates to reading ability in hearing children (Kyle et al., 2016).

Given the relationship between speechreading and reading in deaf individuals, it is important to understand what makes a good speechreader. Although speechreading skill is highly variable between individuals, it is not clear what accounts for this variation or what strategies may be advantageous. A case study with a deaf, skilled speechreader showed that she looked at the mouth during visual speech perception tasks (Lansing & McConkie, 1994), supporting the idea that mouth focus improves access to phonetic information in visual speech. However, gaze direction during visual speech perception has not been studied widely in deaf adults or children.

In the current study we investigated whether children who are born deaf, and therefore are likely to have experienced a greater dependence on visual speech throughout their lifetime, access visual speech in a different way from hearing children. We used eye tracking with children born moderately to profoundly deaf and hearing children ages 5–8 years old while they watched videos of silently spoken sentences. We aimed to address three questions: (1) Do deaf and hearing children differ in the time spent looking at the mouth during visual speech perception? (2) Does the time spent looking at the mouth during visual speech relate to visual speech perception ability in deaf and hearing children? (3) Does the above relationship differ between deaf and hearing children?

## Method

### Participants

Thirty‐three children (20 males) born moderately‐to‐profoundly deaf were recruited from specialist schools for deaf children and mainstream schools with and without hearing impairment units. Eye‐tracking calibration was not possible with two deaf children. Therefore, eye‐tracking data could not be collected from these participants. A further two children were excluded due to having fewer than eight trials with more than 50% tracking. This left 29 deaf participants (18 males). All of the deaf participants were part of a larger study involving a randomised controlled trial of a computerized speechreading training program (Pimperton et al., 2017). The experimental group was trained on a speechreading and reading training computer game and the control group was trained on the same game with number and maths content. The training took place for 10 minutes a day for 4 days a week over 12 weeks, making a total of 8 hours of training. They were then followed up immediately, at 3 months and at 10 months after the end of the intervention. The data used in this study were collected at the final time point. Fifteen of the children in the current study were in the speechreading‐training group and 14 were in the maths‐training group (control group).

All deaf participants had bilateral hearing loss. The average loss in the better ear was 90 dB (*SD* = 27.48 dB; range: 37.5–120 dB; data available for *n* = 20; for *n* = 17, hearing loss in the better ear was 60 dB or greater). Ten children had CIs bilaterally, 17 had hearing aids bilaterally, 1 child had no aiding, and 1 child had one hearing aid and one CI. Five of the children used only British Sign Language (BSL) in the classroom, 11 of them used a mixture of speech and sign, and 13 of them used spoken English only. They had a mean age of 7 years 2 months (*SD* = 7.7 months).

A control group of 59 hearing children (32 males) were recruited from two mainstream schools in Cambridgeshire. Twenty‐nine children (18 males) were selected from this group to be matched to the deaf children in age and gender. Of these 29, 21 children were monolingual English speakers, 5 spoke an additional language at home but had learned English from birth, and the remaining 3 children had been learning English for an average of 3 years (*SD* = 2 years; range: 1–5 years), as reported by their parents. Despite this, these three children had an average standard score of 48 on the British Ability Scales Word Definitions subtest (group range: 42–51); therefore, they were included in the study. The hearing group of participants had a mean age of 6 years 11 months (*SD* = 5.8 months).

The children's scores on speechreading, reading, nonverbal IQ, and vocabulary are shown in Table 1. There were no significant differences between the groups on the Test of Child Speechreading (TOCS) words subtest or the York Assessment of Reading Comprehension (YARC) early word reading subtest. For the YARC early word reading subtest, the lack of difference was due to ceiling effects in both groups. However, the hearing children had significantly higher scores on another test of reading, the YARC single‐word reading subtest, and the British Ability Scales third edition (BAS3) matrices subtest, as shown in Table 1.

**Table 1:** Participant scores on measures of speechreading, reading comprehension, general ability, and vocabulary

		Deaf		Hearing				
Test	Subtest (max range)	Mean (*SD*)	Range	Mean (*SD*)	Range	t(*df*)	*p*	*d*
Test of Child Speechreading	Words (0 to 15)	8.62 (*2.57*)	3‐12	7.45 (*2.72*)	2‐12	1.69 (*56*)	.111	0.42
York Assessment of Reading Comprehension	Early Word Reading standard score (69 to 131)	99.38 (*14.89*)^a^	69‐125	103.31 (*9.38*)	79‐115	‐1.15 (*41.3*)^b^	.254	0.31
	Single Word Reading standard score (69 to 131)	91.07 (*14.34*)	69‐131	104.31 (*16.08*)	73‐131	‐3.31 (*56*)	.001	0.87
British Ability Scales	Matrices T‐score (0 to 80)	35.90 (*6.48*)	26‐48	50.86 (*12.82*)	23‐79	–	–	–
	Vocabulary T‐score (0 to 80)	–	–	51.69 (*7.50*)	41‐68	–	–	–
Picture naming	(0 to 74)	65.93 (*3.95*)	56‐74	–	–	–	–	–

*Note. N* = 29; ^a^ *N* = 26 as 3 children were outside the age range for standard scores; ^b^Equal variances not assumed.

### Eye‐Tracking Methods

Eye movements were recorded using a RED250 eye tracker manufactured by Sensomotoric Instruments (SMI, sampling rate 250Hz). The children were seated with their heads approximately 60 centimetres from a laptop screen and tracking was accommodated between 50 and 80 centimetres from the screen. The children were asked to sit as still as possible. Both eyes were tracked but, as is standard practice, data from only the right eye were used. The eye tracker was first calibrated using a five‐point calibration with a smiley face used as the calibration point. The child was asked to follow the nose in the centre of the face. A four‐point validation was then carried out and the calibration process was repeated if necessary. Drift correction trials were placed in between each video stimulus, showing a smiley face in the centre of the screen.

### Offline Measures

#### Speechreading

To assess speechreading ability offline, the TOCS was used (Kyle et al., 2013, https://dcalportal.org/). For the TOCS words subtest the children watched 15 silent videos of a model (7 male, 8 female) speaking a single word. After each video they selected one of four presented pictures to match the word they just saw. Each child was first familiarised with both models by watching silent videos of them saying the days of the week. After the familiarisation each child had three practice trials before the main test began. The task was self‐paced and lasted approximately 5 minutes.

#### Reading

To assess reading ability the YARC early word and single‐word reading subtests were used. For each of these tests the child was given a list of words and asked to read as many as possible. They were allowed to respond in either English or BSL and were awarded a point for each item they labelled correctly.

#### Vocabulary

The deaf children's vocabulary knowledge was assessed using a picture‐naming task. Each child was shown one picture at a time, taken from the training computer game, and asked to give the name in either speech or sign. They were given one point for each picture they could name correctly in either modality. A response was considered correct if it could be identified as the target word, regardless of pronunciation.

The hearing children were not involved in the training study; their vocabulary knowledge was assessed using a standardized measure, the BAS3 Word Definitions subtest. For this task they were read one word at a time and were asked to provide the definition of each word. The test was administered as instructed in the manual.

#### Nonverbal IQ

All the deaf children had completed the BAS3 matrices subtest at the first time point in the randomised controlled trial. This was 16 months before the current data‐collection point. These data are reported in Table 1. The hearing children completed the BAS3 Matrices subtest in the same testing session as when the eye‐tracking data were collected.

### Online Measure

Eye‐tracking data were collected as the children performed the everyday‐questions subtest of the TOCS (Kyle et al., 2013, https://dcalportal.org/). In this subtest each child watched 12 silent videos of a person asking everyday questions such as “How old are you?”. Six were spoken by a male model and six by a female model. The child was asked to watch each video and then repeat as much of the question as they could. They could give their response in either speech or sign and were given a point for each lexical item labelled (maximum score 62).

In between each video a drift‐correction screen was presented with a smiley face in the centre of the screen. The deaf children were asked to look at the smiley face before the next trial was manually triggered. For the hearing children, looking at the smiley face for 1 second automatically triggered the following trial. If it was not triggered automatically, the experimenter continued the experiment manually and made a note of the corresponding trial.

### Data Analysis

The eye‐tracking data were first cleaned by ensuring that the calibration was correct on the drift‐correct trials. If not, the calibration was adjusted by moving the eye marker to the smiley face, changing the calibration for all subsequent trials. Repeated trials where the child was not on task were removed. Any trials (max = 12) where the child was looking at the screen for less than 50% of the speech time were removed. For the deaf children, the average number of retained trials was 11 (range: 9–12). For the hearing children, only one child had one trial removed and the rest were retained.

Three areas of interest (AOI) were created: one that encompassed the whole screen, one that identified the upper face (Eyes), and one that identified the lower face (Mouth). The Eyes and Mouth AOIs were created using equal‐size semicircles with the flat edge of each meeting on the nose, not overlapping. The AOIs were then moved with the video such that their meeting edge remained equidistant from the centre of the eyes and mouth on the image.

Each stimulus video was coded for the onset and offset of visual speech taken as the moments when the lips first moved from a closed position and when the lips returned to that position. The percentage Net Dwell Time (%NDT) was extracted for each of the three AOIs for each trial – for the pre‐speech segment, the speech segment, and the post‐speech segment, respectively. Each video stimulus was a different length so %NDT was used to allow averaging across trials. Regression models were used to address the hypotheses. The speechreading‐training group and maths‐training group were dummy‐coded and entered into the regression models in order to account for mean differences in performance as an effect of training.

#### Social‐Tuning Score

During analysis we noticed a consistent pattern across participants where the child started each trial by gazing at the eyes, then shifted their gaze to the mouth at the speech onset, returning to the eyes at speech offset. We refer to this pattern as the social‐tuning pattern.

To determine whether this strategy was advantageous for speechreading, a scoring system was devised using the following formula: 


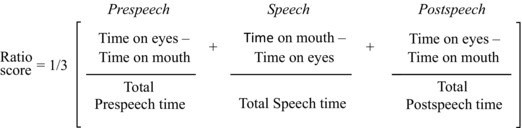


This was used to give a measure of preference for the eyes‐mouth‐eyes pattern observed. This score is referred to as the social‐tuning ratio; a higher social‐tuning ratio reflects greater use of the eyes‐mouth‐eyes pattern when watching the silent videos.

## Results

As the measures were not all normally distributed, bootstrapping was used in all analyses. Equal variances were assumed unless otherwise noted.

There was no significant difference in the number of words correctly identified in the speechreading task between the deaf (*M* = 18.72, *SD* = 14.37) and hearing participants (*M* = 17.45, *SD* = 9.39), *t*(48.2) = 0.40, *p* = .701, *d* = 0.10 (equal variances not assumed). There was no significant correlation between level of hearing loss for the deaf children and the number of words correctly identified in the speechreading task, *r*(18) = −.353, *p* = .127.

### %NDT on the Mouth

#### Group Contrast of %NDT on the Mouth

There was no significant difference in %NDT on the mouth during speech between the deaf (*M* = 64.29, *SD* = 16.89) and hearing participants (*M* = 70.33, *SD* = 16.22), *t*(56) = 1.39, *p* = .161, *d* = 0.36. However, for the deaf children there was a significant difference in %NDT on the mouth during speech between those who did speechreading training (*M* = 70.96, *SD* = 13.08) and those who did maths training (*M* = 57.15, *SD* = 17.99), *t*(27) = 2.38, *p* = .029, *d* = 0.88.

#### Relationship Between Speechreading Scores and %NDT on the Mouth

**Figure 1:**
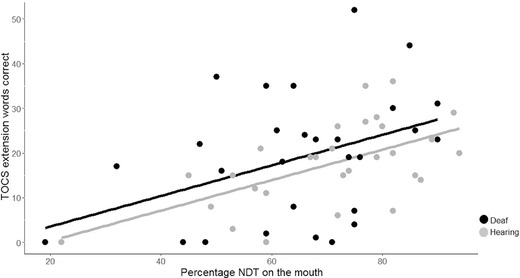
The number of words correctly identified in the Test of Child Speechreading everyday questions task plotted against percentage of net dwell time on the mouth for both deaf and hearing participants. The relationship between these variables was significant for both groups and it is clear that the slopes for the two groups are essentially identical.

There was a significant positive correlation between %NDT on the mouth during speech and the number of lexical items identified in the TOCS extension task for the deaf children, *r*(27) = .399, *p* = .032, and the hearing children, *r*(27) = .586, *p* = .001. The relationships between %NDT on the mouth and speechreading scores for both groups are depicted in Figure 1.

#### Group Differences in the Relationship Between Speechreading Score and %NDT on the Mouth

The relationship between speechreading score and the %NDT on the mouth was significant for both the deaf and hearing groups. From Figure 1 it is also clear that the relationship between these variables is very similar in the deaf and hearing groups. Therefore, a direct contrast was not conducted.

To determine whether the relationship between mouth focus and speechreading performance differed between the deaf participants in the speechreading‐ and maths‐training groups, a multiple regression was calculated to predict the number of lexical items identified in the TOCS extension task based on %NDT on the mouth during speech, the dummy‐coded variable for intervention group, and the interaction between these two variables. The participants’ predicted number of lexical items identified in the TOCS extension task is equal to −5.41 + 0.35(%NDT on mouth) + 16.36(Intervention) − 1.78(Interaction), where Intervention is coded as 0 = Maths, 1 = Speechreading. None of the %NDT on mouth (*p* = .101), Intervention group (*p* = .342), or the interaction term (*p* = .502) were significant predictors of the speechreading scores. When the interaction was dropped from the model the participants’ predicted number of lexical items identified in the TOCS extension task is equal to −1.75 + 0.28(%NDT on mouth) + 4.71(Intervention). The %NDT on mouth was a significant predictor (*p* = .046) while the intervention group (*p* = .434) was not a significant predictor of speechreading scores. As there was no significant interaction between the speechreading‐ and maths‐training groups, the deaf participants can be treated as a single group.

In summary, those in the speechreading‐training group looked at the mouth more (higher mouth %NDT) than those in the maths‐training group. However, there is no difference in how well %NDT predicts speechreading scores between the speechreading‐ and maths‐training groups.

### Social‐Tuning Ratio

Having identified the social‐tuning pattern, we conducted exploratory analyses to test whether it relates to performance on the speechreading task.

#### Group Differences in Social‐Tuning Ratio

We used *t* tests to investigate group differences between the deaf and hearing participants and between speechreading‐training and maths‐training groups in social‐tuning ratio. There was a significant group difference in social‐tuning ratio with hearing children (*M* = .48, *SD* = .14) demonstrating this pattern more reliably than deaf children (*M* = .37, *SD* = .11), *t*(56) = −3.34, *p* = .004, *d* = 0.87. Within the group of deaf children, there was no significant difference in the social‐tuning ratio between those who completed the speechreading training (*M* = .39, *SD* = .12) and those who completed the maths training (*M* = .36, *SD* = .10), *t*(27) = −0.76, *p* = .463, *d* = 0.27.

#### Predicting Speechreading Scores Using the Social‐Tuning Ratio

**Figure 2:**
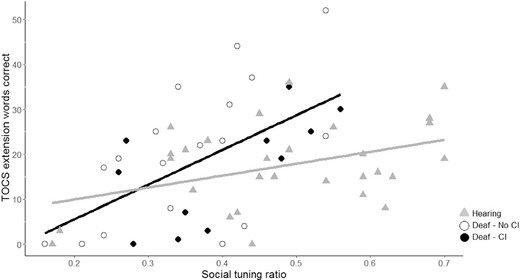
The number of words correctly identified in the Test of Child Speechreading everyday questions task plotted against social‐tuning ratio for both deaf and hearing participants. The relationship between these variables was significant for both groups. The slope for the deaf group (dark red) was steeper than that for the hearing group (black). To illustrate additional aspects of the heterogeneity of the deaf group, deaf children with a cochlear implant (CI) are coded in yellow (*n* = 11) and deaf children without CI are coded in red (*n* = 18). Performance of deaf children with and without CIs are not contrasted statistically due to small sample sizes.

There was a significant positive correlation between the social‐tuning ratio and the number of lexical items identified in the TOCS extension task for the deaf children, *r*(27) = .576, *p* = .001, and the hearing children, *r*(27) = .407, *p* = .028. The relationships between the social‐tuning ratio and speechreading scores for both groups are depicted in Figure 2.

#### Group Differences in the Relationship Between Social‐Tuning Ratio and Speechreading Scores

##### Deaf Group Only (Training Group Contrast)

A multiple regression was calculated for the deaf group only to predict the number of lexical items identified in the TOCS extension task based on social‐tuning ratio, the dummy‐coded variable for intervention group, and the interaction between these two variables. The deaf participants’ predicted number of lexical items identified in the TOCS extension task is equal to −24.68 + 109.57(Social‐tuning ratio) + 29.21(Intervention) − 62.04(Interaction), where Intervention is coded as 0 = Maths, 1 = Speechreading. Social‐tuning ratio was a significant predictor of speechreading scores (*p* = .012), but neither the intervention group (*p* = .072) nor the interaction term (*p* = .184) was a significant predictor. As there was no significant interaction between the speechreading‐ and maths‐training groups, the deaf participants can be treated as a single group. When the interaction term is dropped from the model, the number of lexical items identified on the speechreading task is equal to −11.65 + 72.94(Social‐tuning ratio) + 6.37(Intervention). Social‐tuning ratio (*p* = .003) was a significant predictor of speechreading scores, but the intervention group (*p* = .195) was not.

##### Deaf Versus Hearing Groups

To test whether this relationship differed for deaf and hearing participants, a regression equation was calculated to predict the number of lexical items identified in the TOCS extension task based on social‐tuning ratio, the dummy‐coded variable for hearing status, and the interaction between these variables. The participants’ predicted number of lexical items identified in the TOCS extension task is equal to −9.97 + 77.28(Social‐tuning ratio) + 14.67(Hearing status) – 50.90(Interaction), where Hearing status is coded as 0 = Deaf, 1 = Hearing. Hearing status (*p* = .087) was not a significant predictor of speechreading scores but both social‐tuning ratio (*p* = .001) and the interaction term, *p* = .020, were significant predictors of speechreading scores. This interaction confirms that the slope relating social‐tuning score to TOCS speechreading was steeper in the deaf than the hearing group (see Figure 2).

In summary, the social‐tuning ratio was shown more consistently in hearing than deaf children but related to speechreading scores in both groups, with the deaf children showing a stronger relationship than the hearing children.

#### Predicting Reading Scores Using the Social‐Tuning Ratio

Hearing children used the social‐tuning looking pattern more than deaf children. However, the relationship between this pattern and speechreading accuracy was stronger in deaf than hearing children. We examined this pattern further in exploratory analyses. We reasoned that the social‐tuning pattern may be related to conversational turn‐taking skills, which in turn may be related to other language skills. In our battery we had two common offline measures of reading, the YARC early word and single‐word reading subtests. A composite reading score was calculated by summing the *Z* scores for each of the reading tests. There was a significant positive correlation between the social‐tuning ratio and the composite reading score for the deaf children, *r*(27) = .626, *p* < .001, but not the hearing children, *r*(27) = .273, *p* = .152.

In order to test whether this relationship differed for deaf and hearing participants, a regression equation was calculated to predict the composite reading scores based on social‐tuning ratio, the dummy‐coded variable for hearing status, and the interaction between these variables. The participants’ predicted composite reading scores was equal to −4.06 + 10.92(Social‐tuning ratio) + 2.39(Hearing status) – 7.49(Interaction), where Hearing status is coded as 0 = Deaf, 1 = Hearing. Social‐tuning ratio (*p* = .001) was a significant predictor of reading scores, but Hearing status (*p* = .241) and the interaction term were not (*p* = .086). When the interaction term is dropped from the model, the composite reading score is equal to −2.26 + 6.08(Social‐tuning ratio) − 0.68(Hearing status). In the deaf group alone, Social‐tuning ratio (*p* = .009) was a significant predictor of composite reading scores, but the intervention group (*p* = .177) was not.

## Discussion

Children born deaf must rely to a greater extent on visual input to access spoken language than hearing children. Despite this extensive difference in experience, here we show that young deaf and hearing children do not differ in speechreading accuracy or in the amount of time spent watching the mouth when watching silently spoken sentences (mouth focus). In both groups, mouth focus correlated with the number of words children were able to identify from visual speech and the strength of this relationship did not differ between the deaf and hearing children. In addition, we found that both deaf and hearing children watched the eyes when the model was not speaking but watched the mouth during speech. This gaze pattern correlated with the children's speechreading performance, with a stronger relationship for the deaf than the hearing children. These data provide unique insights into the mechanisms underlying speechreading success in deaf and hearing children. Each of these findings is discussed in detail below.

That we found no difference between deaf and hearing children in their speechreading accuracy is perhaps initially surprisingly. This finding is, however, consistent with previous research showing no speechreading advantage in deaf children over hearing children (Kyle & Harris, 2006; Kyle et al., 2013), despite such an advantage being observed in adults (Mohammed et al., 2006). In addition, deaf and hearing children did not differ in the amount of time spent watching the mouth during silent speech perception. To our knowledge this is the first time this issue has been addressed in children, deaf or hearing. Our findings are in line with previous research showing that hearing adults look at the mouth when auditory information is compromised during audiovisual speech perception in noise and when speechreading (Lansing & McConkie, 2003; Vatikiotis‐Bateson et al., 1998).

For both deaf and hearing children, mouth focus during the silent speechreading perception correlated positively with the number of words correctly identified. Furthermore, there was no difference between the deaf and hearing children in the strength of the relationship between mouth focus and speechreading performance. This suggests that being born deaf and relying on visual speech to access spoken language does not affect how gaze behavior relates to speechreading performance in early childhood. However, studies with adults suggest that developmental changes may be taking place that are yet to be documented. Two previous studies with hearing adults did not find a relationship between mouth focus and speechreading (Lansing & McConkie, 2003; Wilson et al., 2016). Given that there appears to be a speechreading advantage for deaf adults over hearing adults (Bernstein et al., 2000; Mohammed et al., 2006; Pimperton et al., 2017), it is possible that gaze behaviour, and how this relates to speechreading ability, develops differently for deaf and hearing children at some point after the age range tested here, early childhood.

In addition to the predicted relationship between mouth focus and speechreading performance, our analyses revealed a gaze pattern in which the children started each trial looking at the eyes before the speech started, shifted their gaze to the mouth during speech, and returned to the eyes once the speech had finished. Both groups showed this social‐tuning pattern, although the hearing children showed it more consistently than deaf children.

The extent to which children used the social‐tuning pattern was positively correlated with the number of words correctly identified in the speechreading task. This was the case for both deaf and hearing children. These results suggest that those children who shift their attention between the eyes and the mouth when watching someone speak access more information from the spoken message than those who do not. Although causality is not clear from these correlational data, this pattern is consistent with data from hearing adults showing that gaze is task dependent, suggesting that different areas of the face are more relevant for different types of information (Lansing & McConkie, 1999). Indeed, Lansing and McConkie (2003) found the same eyes‐mouth‐eyes gaze pattern identified here when hearing adults watched silently spoken sentences. They suggest that viewers are drawn to the eyes as a high‐contrast stimulus and because they have learned that the eyes express relevant social information, such as the talker's emotions and turn‐taking. Turn‐taking allows conversation to flow without speaking over another person and understanding turn‐taking in conversation is important for language development (Rescorla, 1984). A reduction of gaze toward the eyes during speech has been shown to relate to impairment in language and social understanding in autistic individuals (Hanley et al., 2014). Therefore, the social‐tuning pattern observed here may reflect the deaf and hearing children's understanding of turn‐taking in conversation.

An alternative explanation for the social‐tuning effect is that a moving mouth is a highly salient stimulus and therefore the participant's gaze may be drawn to it (Posner, 1980). However, if exogenous factors were the only explanation, we would expect all participants to watch the mouth for the whole of the speech period. Instead, we observed substantial variation in the time the children spent looking at the mouth and the extent to which they used the social‐tuning pattern. In addition, if eye gaze to the mouth was driven by visual salience alone, this would not explain the relationship we found between the social‐tuning pattern and speechreading performance. Subsequent studies may be useful in directly testing hypotheses regarding the extent to which attention to the mouth during speech is driven by attention to movement.

The relationship between the social‐tuning pattern and the number of words correctly identified in the speechreading task was stronger in the deaf than hearing children. As discussed above, the social‐tuning pattern may reflect the deaf children's underlying language and communication skills. Although visual attention and turn‐taking are important skills for language development in hearing children, they are particularly important for deaf children as they do not have full access to the social cues conveyed through prosody of the voice. We investigated the idea that the social‐tuning pattern relates to other language‐related skills in the deaf children more than hearing children by testing the relationship with reading proficiency. There was a significant relationship between the social‐tuning pattern and single‐word reading scores in the deaf children but not the hearing children. The interaction between hearing status and the social‐tuning pattern was not significant, but the effect size was relatively large (ß = −1.08). Although these analyses were post hoc and exploratory, the trend lends some support to the hypothesis that use of the social‐tuning pattern is a stronger reflection of a deaf child's than a hearing child's broader language abilities. This difference should be investigated in future studies.

An important consideration for the current data set is that the deaf participants were recruited from a large randomised controlled trial, assessing the efficacy of a speechreading and reading intervention. Half the deaf participants received speechreading and reading training and half received maths training. The training groups did not differ in the extent to which they used the social‐tuning pattern. However, children in the speechreading‐training group did spend significantly more time looking at the mouth than those in the maths‐training group. It is not possible to know whether or not gaze behaviour differed between the groups before training because no pretraining eye‐tracking data were collected. However, the mouth was emphasised during the speechreading training as an important location on the face. Therefore, it is possible that the speechreading training may have increased visual attention toward the mouth. Further studies should investigate this hypothesis directly.

The current findings provide insight into how young deaf and hearing children engage with a silently speaking face and how this relates to their speechreading ability. The results suggest that being born deaf, and therefore relying on visual information to access spoken language, does not change the way in which children access visual speech but does affect the extent to which they are able to benefit from employing specific gaze patterns. Although deaf children did not employ the social‐tuning pattern more than hearing children, this pattern was more strongly related to speechreading skills in deaf than hearing children.

Understanding the factors that relate to better speechreading skill in children may inform ways to improve speechreading. In turn, improving speechreading skill in deaf children may lead not only to better spoken language communication skills but also to improved reading skills. Deaf children find learning to read to be a particularly challenging task. Speechreading and reading proficiency have been shown to be positively correlated in deaf children (Arnold & Kopsel, 1996; Kyle & Harris, 2006, 2010, 2011; Kyle et al., 2016). Therefore, understanding any factors that can contribute to improvements in reading are important areas for future research.

## References

[lang12264-bib-0001] Arnold, P. , & Kopsel, A. (1996). Lipreading, reading and memory of hearing and hearing‐impaired children. Scandinavian Journal of Audiology, 25, 13–20. https://doi.org/10.3109/01050399609047550 10.3109/01050399609047550 8658020

[lang12264-bib-0002] Barenholtz, E. , Mavica, L. , & Lewkowicz, D. J. (2016). Language familiarity modulates relative attention to the eyes and mouth of a talker. Cognition, 147, 100–105. https://doi.org/10.1016/j.cognition.2015.11.013 26649759 10.1016/j.cognition.2015.11.013 PMC6367725

[lang12264-bib-0003] Bernstein, L. E. , Demorest, M. E. , & Tucker, P. E. (2000). Speech perception without hearing. Perception & Psychophysics, 62, 233–252. https://doi.org/10.3758/BF03205546 10723205 10.3758/bf03205546

[lang12264-bib-0004] Burnham, D. , & Dodd, B. (2004). Auditory‐visual speech integration by prelinguistic infants: Perception of an emergent consonant in the McGurk effect. Developmental Psychobiology, 45, 204–220. https://doi.org/10.1002/dev.20032 15549685 10.1002/dev.20032

[lang12264-bib-0005] Conrad, R. (1979). The deaf school child: Language and cognitive function. London: Harper & Row.

[lang12264-bib-0006] DiFrancesca, S. (1972). Academic achievement test results of a national testing program for hearing impaired students: United States, Spring 1971 (Report No. 9, Series D). Washington, DC: Gallaudet College, Office of Demographic Studies.

[lang12264-bib-0007] Hanley, M. , Riby, D. M. , McCormack, T. , Carty, C. , Coyle, L. , Crozier, N. , et al. (2014). Attention during social interaction in children with autism: Comparison to specific language impairment, typical development, and links to social cognition. Research in Autism Spectrum Disorders, 8, 908–924. https://doi.org/10.1016/j.rasd.2014.03.020

[lang12264-bib-0008] Jerger, S. , Damian, M. F. , Spence, M. J. , Tye‐Murray, N. , & Abdi, H. (2009). Developmental shifts in children's sensitivity to visual speech: A new multimodal picture‐word task. Journal of Experimental Child Psychology, 102, 40–59. https://doi.org/10.1016/j.jecp.2008.08.002 18829049 10.1016/j.jecp.2008.08.002 PMC2612128

[lang12264-bib-0009] Kyle, F. E. , Campbell, R. , & MacSweeney, M. (2016). The relative contributions of speechreading and vocabulary to deaf and hearing children's reading ability. Research in Developmental Disabilities, 48, 13–24. https://doi.org/10.1007/s13398-014-0173-7.2 26524726 10.1016/j.ridd.2015.10.004 PMC4916263

[lang12264-bib-0010] Kyle, F. E. , Campbell, R. , Mohammed, T. , & Coleman, M. (2013). Speechreading development in deaf and hearing children: Introducing the Test of Child Speechreading. Journal of Speech, Language, and Hearing Research: JSLHR, 56, 416–427. https://doi.org/10.1044/1092-4388(2012/12-0039) 23275416 10.1044/1092-4388(2012/12-0039) PMC4920223

[lang12264-bib-0011] Kyle, F. E. , & Harris, M. (2006). Concurrent correlates and predictors of reading and spelling achievement in deaf and hearing school children. Journal of Deaf Studies and Deaf Education, 11, 273–288. https://doi.org/10.1093/deafed/enj037 16556897 10.1093/deafed/enj037

[lang12264-bib-0012] Kyle, F. E. , & Harris, M. (2010). Predictors of reading development in deaf children: A 3‐year longitudinal study. Journal of Experimental Child Psychology, 107, 229–243. https://doi.org/10.1016/j.jecp.2010.04.011 20570282 10.1016/j.jecp.2010.04.011

[lang12264-bib-0013] Kyle, F. E. , & Harris, M. (2011). Longitudinal patterns of emerging literacy in beginning deaf and hearing readers. Journal of Deaf Studies and Deaf Education, 16, 289–304. https://doi.org/10.1093/deafed/enq069 21307357 10.1093/deafed/enq069

[lang12264-bib-0014] Lansing, C. , & McConkie, G. (1994). A new method for speechreading research: Tracking observers’ eye movements. Journal of the Academy of Rehabilitative Audiology, 27, 25–43.

[lang12264-bib-1001] Lansing, C. R. , & McConkie, G. W. (1999). Attention to facial regions in segmental and prosodic visual speech perception tasks. Journal of Speech, Language, and Hearing Research: JSLHR, 42, 526–539. http://doi.org/10.1044/jslhr.4203.526 10.1044/jslhr.4203.526 10391620

[lang12264-bib-0015] Lansing, C. R. , & McConkie, G. W. (2003). Word identification and eye fixation locations in visual and visual‐plus‐auditory presentations of spoken sentences. Perception & Psychophysics, 65, 536–552. https://doi.org/10.3758/BF03194581 12812277 10.3758/bf03194581

[lang12264-bib-0016] Lewkowicz, D. J. , & Hansen‐Tift, A. M. (2012). Infants deploy selective attention to the mouth of a talking face when learning speech. Proceedings of the National Academy of Sciences of the United States of America, 109, 1431–1436. https://doi.org/10.1073/pnas.1114783109 22307596 10.1073/pnas.1114783109 PMC3277111

[lang12264-bib-0017] Lusk, L. G. , & Mitchel, A. D. (2016). Differential gaze patterns on eyes and mouth during audiovisual speech segmentation. Frontiers in Psychology, 7, 1–11. https://doi.org/10.3389/fpsyg.2016.00052 26869959 10.3389/fpsyg.2016.00052 PMC4735377

[lang12264-bib-0018] Massaro, D. W. (1998). Perceiving talking faces: From speech perception to a behavioral principle. Cambridge, MA: MIT Press.

[lang12264-bib-0019] McGurk, H. , & MacDonald, J. (1976). Hearing lips and seeing voices. Nature, 264, 746–748. https://doi.org/10.1038/264746a0 1012311 10.1038/264746a0

[lang12264-bib-0021] Mitchel, A. D. , & Weiss, D. J. (2014). Visual speech segmentation: Using facial cues to locate word boundaries in continuous speech. Language and Cognitive Processes, 29, 771–780. https://doi.org/10.1080/01690965.2013.791703 25018577 10.1080/01690965.2013.791703 PMC4091796

[lang12264-bib-0022] Mohammed, T. , Campbell, R. , MacSweeney, M. , Barry, F. , & Coleman, M. (2006). Speechreading and its association with reading among deaf, hearing and dyslexic individuals. Clinical Linguistics & Phonetics, 20, 621–630. https://doi.org/10.1080/02699200500266745 17056494 10.1080/02699200500266745

[lang12264-bib-0023] Paré, M. , Richler, R. C. , ten Hove, M. , & Munhall, K. G. (2003). Gaze behavior in audiovisual speech perception: The influence of ocular fixations on the McGurk effect. Perception & Psychophysics, 65, 553–567. https://doi.org/10.3758/BF03194582 12812278 10.3758/bf03194582

[lang12264-bib-0024] Pimperton, H. , Ralph‐Lewis, A. , & MacSweeney, M. (2017). Speechreading in deaf adults with cochlear implants: Evidence for perceptual compensation. Frontiers in Psychology, 8, 106 https://doi.org/10.3389/FPSYG.2017.00106 28223951 10.3389/fpsyg.2017.00106 PMC5294775

[lang12264-bib-0025] Pons, F. , Bosch, L. , & Lewkowicz, D. J. (2015). Bilingualism modulates infants’ selective attention to the mouth of a talking face. Psychological Science, 26, 490–498. https://doi.org/10.1177/0956797614568320 25767208 10.1177/0956797614568320 PMC4398611

[lang12264-bib-0026] Posner, M. I. (1980). Orienting attention. Quarterly Journal of Experimental Psychology, 32, 3–25. https://doi.org/10.1080/00335558008248231 7367577 10.1080/00335558008248231

[lang12264-bib-0027] Qi, S. , & Mitchell, R. E. (2011). Large‐scale academic achievement testing of deaf and hard‐of‐hearing students: Past, present, and future. Journal of Deaf Studies and Deaf Education, 17, 1–18. https://doi.org/10.1093/deafed/enr028 21712463 10.1093/deafed/enr028

[lang12264-bib-0028] Rescorla, L. (1984). Individual differences in early language development and their predictive significance. Acta Paedologica, 1, 97–116.

[lang12264-bib-0029] Smith, N. A. , Gibilisco, C. R. , Meisinger, R. E. , & Hankey, M. (2013, September 11). Asymmetry in infants’ selective attention to facial features during visual processing of infant‐directed speech. Frontiers in Psychology, 4, 1–8. https://doi.org/10.3389/fpsyg.2013.00601 24062705 10.3389/fpsyg.2013.00601 PMC3769626

[lang12264-bib-0030] Sumby, W. H. , & Pollack, I. (1954). Visual contribution to speech intelligibility in noise. The Journal of the Acoustical Society of America, 26, 212–215. https://doi.org/10.1121/1.1907309

[lang12264-bib-0031] Vatikiotis‐Bateson, E. , Eigsti, I. M. , Yano, S. , & Munhall, K. G. (1998). Eye movement of perceivers during audiovisual speech perception. Perception & Psychophysics, 60, 926–940. https://doi.org/10.3758/BF03211929 9718953 10.3758/bf03211929

[lang12264-bib-0032] Wauters, L. N. , Van Bon, W. H. J. , & Tellings, a. E. J. M. (2006). Reading comprehension of Dutch deaf children. Reading and Writing, 19, 49–76. https://doi.org/10.1007/s11145-004-5894-0

[lang12264-bib-0033] Weatherhead, D. , & White, K. S. (2017). Read my lips: Visual speech influences word processing in infants. Cognition, 160, 103–109. https://doi.org/10.1016/j.cognition.2017.01.002 28088039 10.1016/j.cognition.2017.01.002

[lang12264-bib-0034] Wilson, A, H. , Alsius, A. , Paré, M. , & Munhall, K. G. (2016). A tutorial on expository discourse: Structure, development, and disorders in children and adolescents. American Journal of Speech‐Language Pathology, 59(August), 601–615. https://doi.org/10.1044/2016 10.1044/2016_AJSLP-14-0130 27537697

[lang12264-bib-0035] Yi, A. , Wong, W. , & Eizenman, M. (2013). Gaze patterns and audiovisual speech enhancement. Journal of Speech, Language, and Hearing Research: JSLHR, 56, 471–480. https://doi.org/10.1044/1092-4388(2012/10-0288) 10.1044/1092-4388(2012/10-0288) 23275394

[lang12264-bib-0036] Young, G., S. , Merin, N. , Rogers, S. J. , & Ozonoff, S. (2009). Gaze behaviour and affect at 6 months: Predicting clinical outcomes and language development in typically developing infants and infants at‐risk for autism. Developmental Science, 12, 798–814. https://doi.org/10.1111/j.1467-7687.2009.00833.x 19702771 10.1111/j.1467-7687.2009.00833.x PMC2732664

